# Comparative Evaluation of Fracture Resistance of Simulated Immature Teeth Restored with Glass Fiber Posts, Intracanal Composite Resin, and Experimental Dentine Posts

**DOI:** 10.1155/2015/751425

**Published:** 2015-01-01

**Authors:** Vineeta Nikhil, Padmanabh Jha, Akarshak Aggarwal

**Affiliations:** Department of Conservative Dentistry and Endodontics, Subharti Dental College, Meerut, Uttar Pradesh 250005, India

## Abstract

*Aim*. The aim of this study was to compare the fracture resistance of simulated immature teeth restored with gutta-percha, glass fiber posts (GFP), experimental dentine posts (DP) or Intracanal composite Resin (ICR).* Materials and Methods*. Fifty maxillary canines were decoronated, standardized and enlarged until, number 5 Peeso reamers were allowed to simulate immature teeth. After placement of 5 mm of MTA, the canals were divided into 5 groups and filled as follows: Group 1: AH Plus + gutta-percha, lateral compaction; Group 2: GFP luted with PARACORE dual cure resin; Group 3: DP luted with PARACORE dual cure resin; Group 4: PARACORE dual cure resin. A standardized core was built in all groups except in Group 5. Each of the specimens was tested for fracture resistance by universal testing machine.* Results*. The mean fracture resistance were 817 ± 27.753, 1164.6 ± 21.624, 994.4 ± 96.8747, 873.8 ± 105.446 and 493.7 ± 6.945 newtons for Groups 1, 2, 3, 4, and 5 respectively. Independent “*t*” test revealed statistically significant discrepancies, in the fracture resistance among the 4 groups except Group 1 and Group 4 (*P* < 0.05).* Conclusions*. This study suggests that GFP and DP may be preferred for additional reinforcement of immature teeth.

## 1. Introduction

Nonvital, endodontically treated teeth are more prone to fracture against chewing forces than vital teeth [1–3]. Moreover when a developing tooth gets traumatized and becomes nonvital, the development of root also gets halted resulting in divergent dentinal walls of the root canals with open apex. This further increases the susceptibility of these teeth to fracture during normal functional stresses, particularly in the cervical area of the root and often leaving them nonrestorable [4]. Preservation of such teeth is important as extraction may exert adverse effects on nutrition, individual's psychology, and personality development. Additionally extraction followed by implant supported crowns and fixed prosthesis may not be feasible treatment options owing to the ongoing craniofacial growth process in the growing individuals.

Traditionally calcium hydroxide [Ca(OH)_2_] was used to promote the formation of an apical barrier known as apexification which might require a period as long as 5–19 months [5]. But long-term exposure of root dentine to Ca(OH)_2_ may cause adverse effect on properties of dentinal collagen network making it more prone for fracture, thus increasing the possibility of recontamination due to microleakage through the fracture lines [6]. Mineral trioxide aggregate (MTA), a powder aggregate containing mineral oxides, has been recently recommended for apexification. Properties like high pH (12.5 when set), antimicrobial action, biocompatibility, low cytotoxicity, good sealing ability, and ability to set in the presence of blood and moisture are all advantages offered by MTA [7, 8].

Various materials have been verified for increasing the fracture resistance of endodontically treated teeth [9, 10]. Even though glass fibre post (GFP) looks promising [11], a 10-year evaluation study for GFP supported endodontic restorations has reported a high annual failure rate [12]. Recently a few reported cases that used dentin as a post have shown successful outcomes [13, 14]. A preliminary* in vitro* study proved that endodontically treated teeth restored with dentin post (DP) exhibited better fracture resistance than those restored with GFP [15]. This potential of human dentin to serve as post material needs to be examined further.

Hence, the aim of the study was to compare the fracture resistance of simulated immature maxillary canines restored with 4 different types of filling system, gutta-percha and AH Plus, GFP, experimental DP, and intracanal composite resin (ICR) after a 5 mm apical MTA placement.

## 2. Materials and Methods 

Fifty freshly extracted maxillary canines with anatomic crowns similar in dimensions, measuring 6.0 ± 0.5 mm mesiodistally and 8.0 ± 0.5 mm buccolingually, at the cement-enamel junction (CEJ) were selected. The extracted teeth were observed under 8x magnification with the help of dental operating microscope (Gippon Inc., Japan) to exclude the teeth having cracks, caries, and resorptions. Length of each root was standardized to 17 mm by removing the coronal portion (3 mm coronal to the CEJ) and apical portions. Root canal instrumentation was performed using rotary ProTaper Files (Dentsply-Maillefer, Konstanz, Switzerland) as per manufacturer's instructions. For the simulation of teeth with immature apices, Peeso reamers between number 1 and number 5 were introduced in the root canals, and a number 5 Peeso reamer was allowed to protrude 1 mm beyond the apex. The root canals were irrigated using 2 mL of 5.25% sodium hypochlorite after each file and a final flush with 5 mL of 17% EDTA to remove the smear layer. Finally, the root canals were flushed with distilled water and dried using paper points (Diadent, Diadent Group, International, Burnaby, BC, Canada). Ca(OH)_2_ paste (Pulpdent, Watertown, MA, USA), used as an intracanal medicament, was introduced in the root canals using lentulo spirals (Dentsply-Maillefer, Konstanz, Switzerland). After 7 days the calcium hydroxide was retrieved using 17% EDTA in combination with ultrasonic agitation for one minute as recommended by Nandini et al. [16].

Orifices of the root canals were sealed with a temporary restorative material (Cavit, 3 M, ESPE, USA). All samples were incubated for 7 days at 37°C under 100% humidity. Following 7-day incubation period, the Ca(OH)_2_ paste was removed and a 5 mm apical plug of Pro-Root MTA (Dentsply Tulsa Dental, Tulsa, OK) was placed in each tooth with a MAP System (Roydent, Johnson City, TN). The thickness and homogeneity of the MTA plugs were radiographically confirmed. The apices of the experimental teeth were covered with a wet cotton pellet and the samples were kept for 12 h at 37°C under 100% humidity. The remaining portions (12 mm in length) were adjusted with Reforpost drill Size 3 so that they could incorporate the GFP with tip diameter of 1.5 mm (Reforpost, Angelus, Londrina, Brazil). The prepared spaces were rinsed using distilled water and dried using paper points. The samples were randomly divided into five equal groups. 

*Group 1 (Gutta-Percha and AH Plus Sealer)*. Master cones were adjusted and radiographically confirmed. AH Plus sealer (De Trey, Switzerland) was mixed and obturation was completed following lateral compaction technique. Excess materials were sheared off and condensed 1 mm below the canal orifices. The orifices of the root canals were filled using a dual cure composite filling material PARACORE (Coltene-Whaledent, USA) and light cured. 

*Group 2 (GFP and PARACORE)*. Glass fibre post number 3 was tried and radiographically verified. GFP was luted in the canal with PARACORE according to the manufacturer's instructions and light-cured; that is, the nonrinse conditioner was applied into post space of the root canals using a microbrush and rubbed for 30 s. Excess of the conditioner was dried using paper points and a gentle steam of air for 2 s. One drop of adhesive A was mixed with one drop of adhesive B and was applied on the prepared post space using a brush and rubbed for 30 s. Excess adhesive was dried using paper points and a gentle steam of air for 2 s. PARACORE was dispensed directly from the syringe into the prepared post space using the root canal tip, and also the post was coated with PARACORE material. Thereafter, the post was inserted into the post space using gentle pressure and was light cured for 30 s. 

*Group 3 (DP and PARACORE)*. Extracted roots were trimmed to the size of GFP number 3 to fabricate DP. The impression of the glass fibre post number 3 was made using silicone impression material (Soft Putty, 3M ESPE) and a mould was created using clear acrylic. The trimmed dentin posts were tried to fit in the mould to standardize the size of the dentin posts. They were autoclaved for 40 minutes at 240°F and 20 psi [17], and entire procedure of Group 2 was repeated for luting DP to the root canal space with PARACORE according to the manufacturer's instructions. 

*Group 4 (Backfill with PARACORE)*. The post spaces were filled with PARACORE according to the manufacturer's instructions using automixing tips from apical to coronal direction and light cured. 

*Group 5 (Control)*. The remaining portions of root canals were left unfilled. The orifices of the root canals were filled with PARACORE and light cured.

Standardized cores were built with PARACORE according to the manufacturer's instructions on all samples using sterile round plastic straw with a diameter of 5 mm which were cut to achieve a length of 8 mm. All samples were then thermocycled for 500 cycles at 5°C and 55°C with a dwell time of 30 s and transfer time of 5 s. 

*Fracture Testing*. Roots of all specimens were covered with a thin layer of silicone impression (Soft Putty, 3M ESPE) material to simulate the periodontal ligament. The specimens were perpendicularly embedded in self-curing acrylic resin poured in identically shaped cylinders, leaving a distance of 2 mm between the top of the acrylic and the CEJ. Specimens were subjected to a compressive load in a universal testing machine (Instron Corp, Norwood, MA) at a crosshead speed of 1 mm/min, with the use of a jig fabricated to accommodate the tooth/acrylic block assemblies that allowed loading at 135° angle at 3 mm incisal to CEJ on lingual surface. Failure loads were determined and recorded in newtons. Apart from descriptive statistical methods (mean, standard deviation), independent “*t*” test was used for statistical analysis as the number of samples was less than 30 in each group and the groups were independent of each other.

## 3. Results

The mean and standard deviation of the maximum load till fracture for each group is presented in Table 1. Under static loading, Group 2 exhibited the highest fracture resistance (1164.6 ± 21.624 N), followed by Group 3, Group 4, and Group 1 and further by Group 5 at 994.4 ± 96.8747 N, 873.8 ± 105.446 N, 817 ± 27.753,  and 493.7 ± 6.945 N, respectively. Direct pair-wise comparison between different groups by independent “*t*” test which revealed statistically significant discrepancies in the fracture resistance values among the 5 groups (*P* < 0.05) is presented in Table 2. The comparison given in this table shows statistically significant differences between all the compared groups, except between Group 1 and Group 4. Also there was statistically significant difference between the fracture resistance values of control group (Group 5) and the experimental groups (Groups 1, 2, 3, and 4) taken together.

## 4. Discussion

Apexification and root reinforcement might counter the reduced mechanical properties resulting from incomplete root formation. MTA is able to form an immediate apical seal. Placing a thick layer of dentin-bonded intracanal resin composite around a fiber post when required significantly improved the fracture resistance of thin-walled roots [18].

Pertaining to the susceptibility of maxillary anterior teeth to trauma, they were selected for the study. The canals were medicated with Ca(OH)_2_ for 1 week, prior to placement of MTA for apexification, to simulate clinical conditions. As suggested that a post with the same modulus of elasticity as root dentin distributes applied forces evenly along the length of the post [19], three such materials were chosen for fabrication of post. Other than GFP and ICR, DP were fabricated and included in this study because DP might resemble root dentin in all the physical properties and may allow post flexion to mimic tooth flexion so that the post acts as a shock absorber, transmitting only a fraction of the stresses placed on the tooth to the dentinal walls [15, 20]. In addition it may form a biomechanically homogenous unit with the root dentin that results in uniform stress distribution [21].

Cores were not restored with crowns to exclude any external strengthening influence on the post and core foundations. However, extrapolation to a clinical situation cannot be made without the use of crowns; hence this might be a limitation of the study. This may represent possible area for future research.

The results of the present study indicated that control group exhibited significantly lower fracture resistance value compared to experimental groups (*P* < 0.05). The control group was compared with the experimental groups taken together to know whether placement of post did have any effect on the fracture resistance of the teeth. The results of this study show that the placement of post did increase the fracture resistance of the teeth. Both GFP and DP showed statistically better reinforcing effect (*P* < 0.05) on simulated immature teeth than gutta-percha and sealer alone except GR 4 (composite resin post). Although ICR showed higher mean failure load value than gutta-percha and AH Plus sealer backfilling, statistically the difference was insignificant (*P* > 0.05). This finding is in accordance with study conducted by Schmoldt et al. [22] but differs from Lawley et al. [23]. Thermocycling might be one of the reasons for this as thermocycling may significantly reduce the flexural strength of composite resin [24]. Thermocycling would have affected other groups also as dual cure composite resin was used for luting all the post and for core build-up but its effect would have been less as lesser amount of composite resin was required for luting the post when compared to ICR. However,* in vitro* studies are limited in their ability to accurately simulate clinical conditions.* In vivo*, the periodontal ligament and bone might help minimize the temperature differences experienced by the restorative materials. Future studies should evaluate to what extent thermal changes affect composite placed into the root canal space.

In our study, the teeth restored with the GFP were actually stronger than all other groups (*P* < 0.05). The results of this study are in agreement with a study by Schmoldt et al. [22] which showed that fiber posts significantly reinforce the teeth and might decrease the risk of fracture. This finding might be related to a similar modulus of elasticity of fiber posts and dentin and more even distribution of force along the root [21, 22]. In addition, fiber posts have been shown to increase the flexural properties of the core composite surrounding the post [25]. Luting glass fiber post with dual cure glass-reinforced composite resin, Para Core, might have created a monoblock [21].

In the present study reinforcement of simulated root was significantly better (*P* < 0.05) with GFP (1164.6 N) when compared to DP (994.4 N). This result differs from a previous study [15] that concluded reinforcing effect of DP better than GFP. Different study designs used in the two investigations may account for this result. The aforementioned authors did not include simulated immature teeth in their study nor did they conduct thermocycling. They included CAD/CAM to shape the DP; thus they were well fitting but in the present study DP were manually prepared and, therefore, may not be exactly fitting leading to more amount of composite resin material surrounding post, thus being more affected by thermocycling than in the previous study.

Although teeth used in the present study morphologically mimicked immature teeth, they were unable to do so in terms of tissue composition or physical characteristics. Nevertheless, as all the experimental teeth went through the same procedures for the simulation of immature teeth, it can be assumed that they still allow making a relative comparison between materials' reinforcing effects on physically weak dentinal structures.

## 5. Conclusions

Within the limitations of this study, it can be concluded that all GFP, DP, and ICR were capable of exerting a reinforcing effect on simulated immature teeth. The results of the present study should certainly be taken with caution, as in all studies of an* in vitro* nature. Many factors may account for obtaining different results in the clinical environment, for example, occlusion specific to each individual, habits of mastication, parafunctions, and structure of the alveolar bone.

Future studies should be directed to assessing these parameters to draw a clear picture regarding elastic post systems intended to be used for the reinforcement of immature teeth.

## Figures and Tables

**Table 1:** Mean and standard deviation and S.E.M. of forces (N) for different groups.

S. number	Groups	Mean ± S.D.	S.E.M.
1	Gr 1 (AH Plus + gutta-percha)	817 ± 27.753	8.777
2	Gr 2 (fibre post)	1164.6 ± 21.624	6.839
3	Gr 3 (dentine post)	994.4 ± 96.8747	30.6343
4	Gr 4 (Para Core)	873.8 ± 105.446	33.348
5	Gr 5 (control)	493.7 ± 6.9450	2.196

**Table 2:** Pair-wise comparison between different groups for comparing the significant difference in forces.

S. number	Pair of groups	Probable values of independent “*t*” test
1	Gr 1 and Gr 3	0.0002^*^
2	Gr 2 and Gr 3	0.0003^*^
3	Gr 4 and Gr 3	0.0159^*^
4	Gr 5 and Gr 3	0.0000^*^
5	Gr 1 and Gr 2	0.0000^*^
6	Gr 1 and Gr 4	0.1298
7	Gr 1 and Gr 5	0.0000^*^
8	Gr 2 and Gr 4	0.0000^*^
9	Gr 2 and Gr 5	0.0000^*^
10	Gr 4 and Gr 5	0.0000^*^
11	Control (Gr 5) and experimental group (Gr 1 + Gr 2 + Gr 3 + Gr 4)	0.0000^*^

^*^It shows a significant difference at 0.05 level of significance.
